# Implications of COVID-19 for Public Health Theory and Praxis From a Complex Systems Perspective

**DOI:** 10.34172/ijhpm.2022.7349

**Published:** 2022-12-28

**Authors:** Vahid Yazdi-Feyzabadi, Esmaeil Khedmati Morasae, Sajad Delavari

**Affiliations:** ^1^Health Services Management Research Centre, Institute for Futures Studies in Health, Kerman University of Medical Sciences, Kerman, Iran.; ^2^Centre for Circular Economy, Exeter University Business School, University of Exeter, Exeter, UK.; ^3^Health Human Resources Research Centre, School of Health Management and Information Sciences, Shiraz University of Medical Sciences, Shiraz, Iran.

## Introduction

 Complex systems, in a nutshell, are dynamic networks of interactions and interdependencies among a remarkable number of adaptive agents (eg, human beings and social institutions). According to this dynamic approach all entities are interrelated and have mutual effects on each other. This is in contrast with static approach when the failure of one entity has little to no effect on the system as-a-whole. Such dynamic networks of relations give rise to new properties at different scales of the system.^1^ In a complex system, actions in the environment to achieve a particular goal can change other agents’ goals and behaviour, which in turn could create unintended side-effects.^2,3^ For instance, unjustified and unreasonable inhumane interventions in the ecosystem cycles have led to the elimination and endangerment of elements of cycles in many cases; some of these elements had pivotal functions in the cycles but have gone missing. This vacuum in the cycles is already having adverse effects in the world and we can see traces of such effects. For example, due to excessive use of pesticides in agriculture, some insects have started going extinct and some diseases like cancers or emerging infectious diseases are on the rise. Such catastrophes oblige us to consider how systemic disruptions in ecosystem cycles can lead to whole system dysfunctions. The public health implications of such systemic dysfunctions are being increasingly addressed in the literature.^4-6^

 According to this systemic approach, this conclusion could be right that humankind is the prime suspect in such grave ecological and public health catastrophes. In fact, studies have found that infectious diseases are significantly correlated with ecological factors^7^ and human interactions with the natural world is a critical issue in emergence of novel pathogens.^3^ We are to use the systemic approach to ecology, infectious diseases, and humanity’s role and show what a complexity perspective can tell us about coronavirus disease 2019 (COVID-19), in specific, and public health theory and praxis, in general.

## Hyper-connectedness, Globalization, Health Threats

 From a complex systems perspective, globalization can be considered as a process of increasing the complexification of social and economic systems by cross-linking them.^8^ The problem such cross-linking causes is twofold: first, the move from an individual or a local scale to a global scale becomes hugely shortened. The COVID-19 virus effectively used such a shortened intra-scale connectivity to spread very quickly; a seemingly unimportant eating behavior of a person in a remote area in China shook the whole world in a matter of days. This movement is more problematic when we face phenomena with exponential growth rate (eg, COVID-19 virus).^9^ Second, globalized hyper-connected networks of supply chains cannot be optimized effectively and any shock can propagate and cripple the whole network, posing huge existential risks for everybody in the network.^10^ COVID-19 has already created havoc in our supply chain networks.^11^ It is not difficult to think of second-order effects of such a shock, especially in terms of huge unemployment rates that can be as dangerous to public health as the COVID-19 virus. Therefore, as complex systems research shows that hyper-connected networks are more sensitive to shocks and quite brittle, we have to plan for more of global-scale problems in public health, at local and global levels, from now on. However, according to Gao et al, a highly connected network may provide a link redundancy that makes the system more robust.^12^ Therefore, the creation of collective intelligence at a global scale might be a solution that needs huge diplomatic efforts and collaborations.

## Centralism, Localism, Public Health

 There has been a strategy in public health that “think globally but act locally.”^13^ Considering the evidence that hyper-connectivity makes systems fragile,^14-16^ it seems that we better change such a strategy to “think locally, act locally, but collaborate globally.” Complex systems theory favors localism in public health as this issue can lead to four favorable outcomes. First, centralization swamps when it faces the complexity of context, while a localism approach in public health embraces and adapts to the complexity of each local place. Second, context gains its due traction in complexity-informed localism. The context in which a system embeds is of huge importance in complexity science as part of the dynamicity of a system comes from the context. Complexity-driven localism re-directs attention to peculiarities of context in public health and that every measure should be context-sensitive or embrace the failure. This matter can also be a remedy to the replication crisis in public health that emanated from blindness to context and complexity.^17^ Third, sensitiveness to the context in complex systems leads naturally to the participation of local people in every action to intervene in the system and context. To better put, complexity theory invites collective sense-making to better understand the complexity of a system and the interdependence of problems and solutions.^18^ This collective sense-making is more possible at local levels where people have had skin in the game in that context for most of their life.^19,20^ Fourth, local supply chains with fewer global out-goings and in-comings are more robust and can survive volatilities of any sudden change, ensuring economic system resilience in harsh times.^16^ All this invites us to local and community-level management of COVID-19 and its economic repercussions while collaborating at national and global levels.

## Ecological Complexity, Viruses, Public Health

 According to complex systems theory, complexification of any system comes at a price: reduction in complexity of the ecology that the systems is embedded in.^21^ The complexity of human societies has hugely increased over the past centuries and this has come at the price of reduction in complexity of the natural environment where human societies get their required resources and energy from.^21^ If this path of increase in the entropy of ecological systems continues, a more volatile future and even a chance of collapse should be expected that have devastating effects on public health. Moreover, diminishing the complexity of ecology has created another problem in terms of throwing more viral pandemics at human systems. Ecological systems have a graceful and slow rate of change if left alone, but human activities have speeded up the change. Therefore, because of destructions, new ecological niches have been being created with a faster rate where only a couple of species like viruses, that have fast rates of evolution, can cope with. The destructions give a selective advantage to viruses to adapt to the new niches where most of the species are doomed. The virus then uses new species in the created niche to adapt and survive. As a result, we see that new diseases and epidemics are emerging continually.^22^ Therefore, preserving the complexity of ecological systems and aligning the complexity of the human system under that complexity should be a must in public health theory and praxis to save us from frequent epidemics and pandemics.

## Uncertainty, Efficiency, Resilience

 Even if we embrace localism and prioritize ecological complexity conservation, we still need to be open to uncertainty and volatility in complex systems. In fact, due to interdependencies and non-linearity of relationships within and across complex systems, unpredictability and uncertainty are of bold features of all complex systems.^23^ COVID-19 is a good example of such unpredictability. Our health systems, on the contrary, are designed in a way that they overlook uncertainty and volatility and praise certainty. For instance, efficiency is of greater importance to these systems than robustness, resilience, and anti-fragility and this issue haunts us when an unpredictable event strikes. Resilience indicates an adept capacity to re-arrange structures and learn in response to system disturbances rather than only maintaining functionality (robustness) in response to unexpected events.^24^ Planning simultaneously for uncertainties and certainties is a better way to manage complex systems in public health.

## Constraints, Coherency, Policies

 Relationships and interdependencies among agents in complex systems can be understood as constraints. The agents in a complex system constrain each other in circular ways, creating feedback loops of constraints within and across scales of the system. These constraints, however, are enabling constraints as they expand the probability space for a complex system to navigate and dispose of agents that are not able to do so on their own. To be more precise, the enabling constraints create the required coherency in the systems so that its agents can collectively decide and act.^25,26^ The coherency is stronger at local community levels, allowing for mobilization of the agents is a system to better respond to stressors, eg, COVID-19, at more local levels. More importantly, policies can be understood as a specific kind of constraints in complex systems. There are lots of constraints in complex systems that can directly or indirectly affect policies and coherency of systems. However, seeing public health policies as constraints in a complex system can pose new questions regarding the possibilities that they create for the system, their effects on the coherence of the system, effects of other constraints on policies, and effects of policies on other constraints. These are the new question and ways of thinking in terms of policy-making in public health that complexity science can offer. For instance, specific policies of COVID-19 management in different countries can be seen from the way they constrain the agents in those policies and the possibilities that they created in this regard.

## Complexity Science, Consilience, One Health

 Complexity science invites *consilience* as part of its methodology by which the same issue or problem is approached from different angles and by various scientific disciplines for investigation and understanding. The result is a more holistic understanding of the problems and possible solutions.^27,28^ This specifically applies to COVID-19 for which a wide range of scientific disciplines from humanities to physics, and virology have to come together and collaborate across local, national, and global levels to solve the problems of the pandemic. However, due to the systemic nature of most public health problems, consilience should be a norm in public health research and praxis. Interestingly, this view is in line with one health approach where collaborative efforts of multiple scientific disciplines are invited to work locally, nationally, and globally to ensure an optimal level of health for human beings, animals, and ecosystems as members of a delicate complex system.^29^ Interestingly, complexity science shares more with one health approach as this approach quite systemically holds that any disturbance in the cycles of interdependencies of the ecological system, especially by the destruction of habitats, can lead to malaises (eg, pandemics) across the whole system. Therefore, complex systems science and one health approaches call for public health programs, studies, policies, and measures that concurrently consider such delicacies of complex ecological systems at local, national, and global levels.

## Panarchy, Ecology, Public Health Theory

 However, apart from similarities between complexity science and one health approaches, complexity science offers some theoretical frameworks that better capture the dynamicity of the ecological systems and their relationships with human systems. Public health theory can adopt such frameworks to re-invite the ecology into public health. Panarchy framework in complexity science, specifically, illuminates how socio-ecological systems at local, national, and global scales transform.^30-32^ According to this framework, complex socio-ecological systems can be understood as spatially-nested adaptive cycles that temporally move through four recurring transformation stages (Growth, Reservation, Collapse, and Reorganization) (Figure). The speed of transformation is faster at local (low) scales and can reverberate to higher scales (cross-scale linkages), leading to perturbations at all scales. The speed of transformation is slow at upper scales but can cascade to the lower scales. Growth level is the stage where there are lots of opportunities for new connections and linkages to emerge between system agents (human beings and ecological elements), resources are not used (there is redundancy), and the system is quite resilient to shocks. Conservation is the stage where there is a huge number of fixed connections (hyper-connectivity), efficiency is high (lack of redundancy), and the system is very brittle and non-resilient to shocks. Collapse is the stage where a system could not adapt to a shock/crisis and connections and cohesion of the system are disrupted. The reorganization is the stage where the system recovers from the crisis and there is a plethora of possibilities and potentials for the system and it can enter the next stage of growth to re-start the adaptive cycle.

**Figure:**
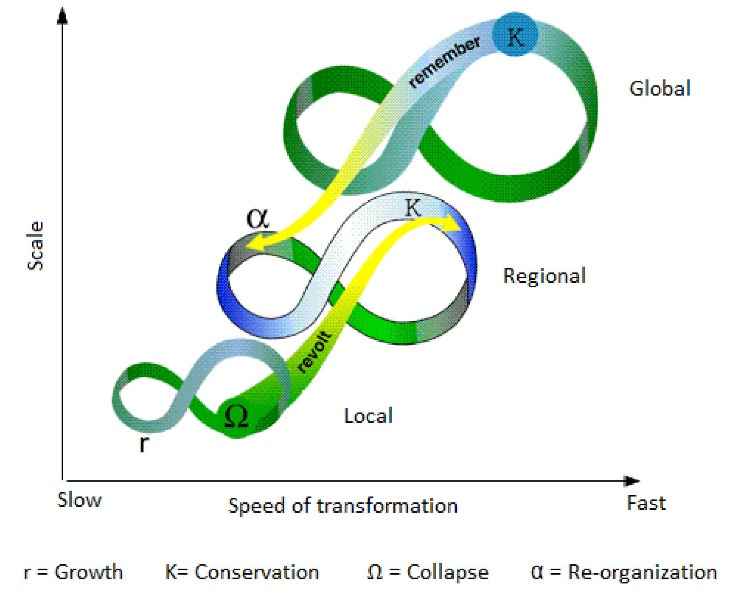
A Schematic Illustration of the Panarchy Adaptive Cycles and its Stages and Cross-linkages.^33^

 COVID-19 virus was part of a local socio-ecological system going through the adaptive cycle on which the system sits somewhere between growth and conservation stages, exploiting all the possible connections and resources (adding to the entropy of the ecology) and becoming less resilient. At the same time, the wider regional and global socio-ecological systems have been sitting in the conservation stage for a long, being more efficient and brittle. Therefore, sudden turbulence in the local socio-ecological system, by the introduction of the COVID-19 virus to the human system, cascaded to regional and global scales and created a disruption and crisis at all scales. This outlook can be adopted for other public health problems as well where ecology becomes an important issue for the public’s health.^30^ One specific point that should be taken into account about COVID-19 pandemic is that the national trends tend to aggregate and hence smooth the patterns happening at local scales. So, the local epidemics are starting and ending faster than the state level with the regional, national, and global trends following increasingly slower trends (just like the changing tempo the Panarchy framework proposes). In other words, the trend of the pandemic could be different at each level and may also be different by disease. Therefore, there is a striking contradiction between the dynamics of the pandemic at multiple scales that we have observed and the slow-fast distinction organized by scale.

 In addition to a systemic outlook to cross-scale socio-ecological systems and their transformations, the Panarchy framework also offers some strategies to make the systems more resilient against shocks and crises. To be precise, the framework invites for identification of variables and feedback loops at each scale that has a slower rate of change, variables and feedbacks like biodiversity, depletion of ecological resources, and climate trends. Changes in these slow variables and feedbacks are quite subtle and slow and need meticulous investigation and effort to capture. However, any irregular change in such variables indicates that the system is stepping towards the crisis and collapse phase and the intricate relationships in the system are beginning to disentangle and ecological entropy is increasing. Therefore, managing slow variables and feedback becomes a priority across the scales. Moreover, the Panarchy framework calls for considering and ensuring redundancy and diversity in human and ecological systems. Too much focus on efficiency in healthcare and business systems (eg, supply chains) makes these systems fragile in crisis time as there is not enough redundancy, in terms of human and material resources, to manage and weather off the crises (eg, pandemics) effectively. The reduction of diversity in the ecological systems also makes these systems fragile and disintegrable. Therefore, redundancy and diversity should be added to the list of socio-ecological management across various scales.

 We lack such an outlook on ecological systems, human systems, their integration, dynamics, and resiliency, offered by the Panarchy framework, in public health science. It is hoped that the adoption of such frameworks can better equip public health practitioners and scientists with insights to see the links between ecology and public health and plan for the health of socio-ecological systems on the same planning sheet. This matter also opens up the opportunities to collaborate across scales, though the localism should be still at the centre of the public health programs considering the manageability of the complexity of socio-ecological systems at local scales.^32^

## Conclusion

 To wrap up, in terms of COVID-19 and public health, complexity thinking informs us that hyper-connectivity of the globalized world makes human systems more fragile, especially for pandemics, and we have to plan for it; localism should be encouraged as it makes complexity more manageable at local scales; uncertainty should be befriended and planned for; ecological systems should re-join public health theories and praxis especially through newer theories and frameworks.

## Ethical issues

 Not applicable.

## Competing interests

 Authors declare that they have no competing interests.

## Authors’ contributions

 SD and VYF conceptualize the idea and write the first draft of the manuscript. EKM critically revised the manuscript.
